# Spontaneous Regeneration of Human Photoreceptor Outer Segments

**DOI:** 10.1038/srep12364

**Published:** 2015-07-27

**Authors:** Jonathan C. Horton, Alicia B. Parker, James V. Botelho, Jacque L. Duncan

**Affiliations:** 1 Beckman Vision Center University of California San Francisco San Francisco, California, 94143, USA

## Abstract

Photoreceptors are damaged in many common eye diseases, such as macular degeneration, retinal detachment, and retinitis pigmentosa. The development of methods to promote the repair or replacement of affected photoreceptors is a major goal of vision research. In this context, it would be useful to know whether photoreceptors are capable of undergoing some degree of spontaneous regeneration after injury. We report a subject who lost retinal function in a wide zone around the optic disc, giving rise to massive enlargement of the physiological blind spot. Imaging with an adaptive optics scanning laser ophthalmoscope (AOSLO) showed depletion of cone outer segments in the affected retina. A year later visual function had improved, with shrinkage of the enlarged blind spot. AOSLO imaging showed repopulation of cone outer segments, although their density remained below normal. There was a one-to-one match between sites of formerly missing outer segments and new outer segments that had appeared over the course of the year’s recovery. This correspondence provided direct morphological evidence that damaged cones are capable, under some circumstances, of generating new outer segments.

Rods and cones are specialized neurons, containing stacks of membrane laded with photopigment in a compartment called the outer segment, which is anchored by a cilium to the rest of the cell. They initiate the process of vision by converting patterns of light energy into graded electrical potentials. Diseases that affect photoreceptors are a major worldwide cause of vision loss. In 1988, a new syndrome called acute idiopathic blind spot enlargement (AIBSE) was described^1^. It consists of sudden onset of photopsia accompanied by a scotoma, or localized area of vision loss, surrounding the blind spot^2^. The ocular fundus remains normal on clinical examination but electroretinography shows impaired function of photoreceptors. Subsequently, AIBSE has been linked to a family of enigmatic photoreceptor diseases, thought to be infectious or autoimmune, including acute zonal occult outer retinopathy (AZOOR), acute macular neuroretinopathy (AMN), multiple evanescent white dot syndrome (MEWDS), and multifocal choroiditis. In these conditions, known collectively as the White Dot Syndromes or AZOOR complex disorders, visible fundus findings are often subtle or absent^3^,^4^,^5^. However, optical coherence tomography has shown morphological changes in the retinal layer containing photoreceptor outer segments^6^,^7^,^8^,^9^,^10^.

In these rare photoreceptor diseases, there is usually a substantial degree of vision recovery. The cellular events underlying this gradual improvement are obscure. One possibility is that some lost or damaged outer segments are replaced following photoreceptor damage.

This study was conducted to determine whether, after injury, photoreceptors have the capacity for spontaneous regeneration of new outer segments. To address this issue we performed serial imaging using an adaptive optics scanning laser ophthalmoscope (AOSLO) in a human subject with a dense but transient visual field defect from AIBSE. Adaptive optics, by compensating for optical imperfections, allows one to resolve individual cones in the intact eye^11^,^12^. The technique relies upon the reflection of light back to the instrument by cone outer segments^13^. If outer segments are missing, they appear as dark gaps in AOSLO images of the photoreceptor mosaic. By imaging during the phase of acute vision loss, and later after visual improvement, one can determine if the population of cone outer segments has been replenished.

## Results

**Figure 1:**
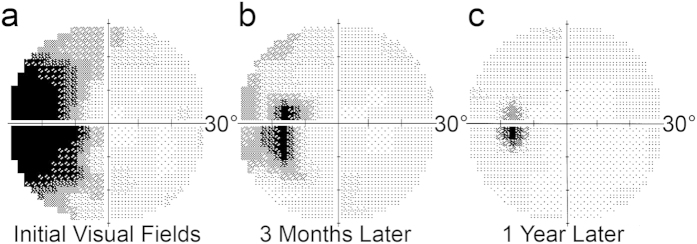
Onset and resolution of AIBSE. **(a)** Map of the central 30° of the visual field in the left eye plotted a month after the development of vision loss showing an enormous blind spot. The grayscale depicts retinal sensitivity, with black representing no response to the brightest stimulus generated by the perimeter. **(b)** Three months later, the scotoma has shrunken. **(c)** A year later, there is further improvement, although the blind spot is still twice normal size.

**Figure 2:**
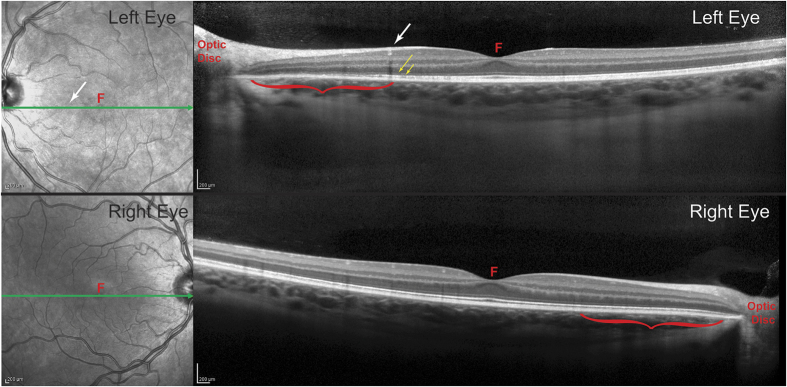
Spectral domain optical coherence tomography shows damage to the photoreceptors in the nasal retina of the left eye. Each cross-section corresponds to the green line in the adjacent fundus image. In the left eye, it passes through the blood vessel (white arrow) visible in Fig. 3, marking the eccentricity at which the density of cone outer segments plummets and the absolute scotoma begins. Between the white arrow and the optic disc (red bracket), the outer nuclear layer in the left eye appears slightly thinner than at the corresponding location in the right eye (red bracket). In the left eye, the dim line representing the external limiting membrane (long yellow arrow) is preserved, but the bright line marking the junction of the inner and outer segments (short yellow arrow) becomes disrupted. Critically, there is substantial volume loss in the layer containing outer segments, which is normally seen as a gray band just to the scleral side of the inner/outer segment junction. F = fovea.

An 18-year-old woman experienced flashing lights in the temporal field of her left eye for several weeks, along with onset of a dense scotoma. Although her acuity was normal, visual field testing (Fig. 1a) revealed massive enlargement of the physiological blind spot. A multifocal electroretinogram (ERG) showed marked reduction of electrical potentials in a region corresponding to the scotoma (Fig. S1). In addition, optical coherence tomography showed focal attenuation of the photoreceptor layer, beginning at the border of the scotoma (Fig. 2). The outer nuclear layer, which contains cone cell bodies, was only slightly reduced in thickness. However, there was collapse of the layer containing the outer segments. The subject’s symptoms, enlarged blind spot, reduced ERG, and disrupted photoreceptor layer visible on optical coherence tomography all supported strongly the diagnosis of AIBSE^14^.

**Figure 3:**
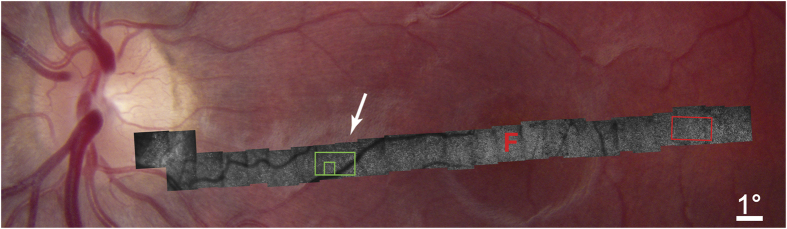
AOSLO imaging of left retina showing loss of cone outer segments. The retina appears normal in the fundus photograph, but extensive loss of outer segments is revealed in the superimposed montage of AOSLO images. Dropout is visible everywhere in the AOSLO montage, but increases sharply at 6.5° (arrow) from the optic disc coinciding with the border of the subject’s enlarged blind spot. Arrow indicates blood vessel marked in Fig. 2. F = fovea. For a higher resolution image, see Fig. S2. Red boxed region is shown in Fig. 4, green boxed region in Fig. 5a,b.

With a conventional ophthalmoscope, no abnormality could be detected in the appearance of the optic disc or retina in the left eye. Faced with compelling evidence of outer retinal dysfunction, AOSLO was performed to investigate the morphology of individual photoreceptors in the vicinity of the optic disc. Images were acquired along a 21° horizontal swath, which began temporally in the retina (8°), passed through the fovea (0°), and ended nasally at the optic disc (12.75°) (Fig. 3). For comparison, imaging of the right retina was also performed (Fig. S5).

**Figure 4:**
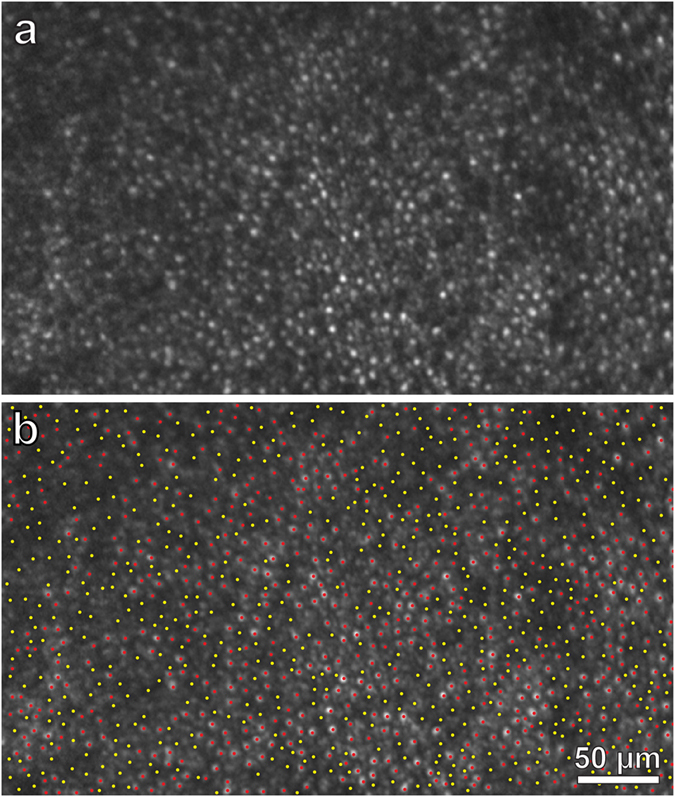
AOSLO image shows patchy loss of cone outer segments in left temporal retina, far from retina corresponding to the enlarged blind spot. **(a)** Zone centered at 6.5° temporal to the fovea (red box, Fig. 3), at a mirrored location to the box in Fig. 5a, showing many absent cone outer segments. **(b)** Identification of 549 missing (yellow dots) and 650 intact (red dots) outer segments in (**a**). The region measures 0.10 mm^2^, yielding a total density of 11,990 cones/mm^2^, with 6,500 intact cones/mm^2^ and 5490 missing cones/mm^2^. The density in the normal right retina at the same eccentricity is 12,290 visible cones/mm^2^ (Fig. 5e,f). Despite loss of nearly half the cones, retinal sensitivity was depressed by only a half log unit (3–6 dB) (Fig. S3).

Although we expected to find decimated photoreceptors in the nasal retina surrounding the optic disc, to our surprise AOSLO imaging showed that even in the temporal retina the photoreceptor mosaic was highly abnormal. It was punctuated by dark holes, that presumably represented cones with damaged or absent outer segments. Often, these diseased cones occurred in clumps, as if the responsible process had spread locally. A sample region centered 6.5° temporal to the fovea was analyzed quantitatively (Fig. 4). Of a total of 1199 cone profiles, 549 (46%) lacked a visible outer segment. The density of photoreceptors with a normal appearance was only 6,500 cones/mm^2^. This finding showed that cone damage in AIBSE can be widespread, occurring at sites located far from the optic disc. Although nearly half the outer segments in this portion of the temporal retina appeared to be absent, there was only a 3–6 decibel (dB) loss of retinal sensitivity in this region on visual field testing (Fig. S3). This was not sufficient to produce a noticeable scotoma.

**Figure 5:**
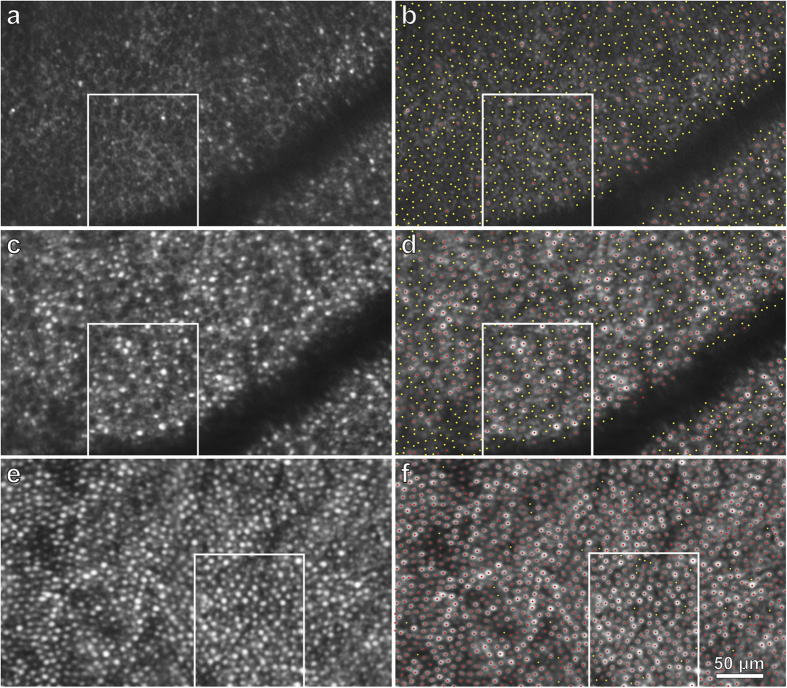
AOSLO shows recovery of cone outer segments in the left eye. **(a)** Retinal zone centered at 6.5° nasal to the fovea (see green box, Fig. 3) imaged 6 weeks after scotoma onset, showing a field of dark, circular profiles that are presumably absent outer segments. There is a smattering of intact cones, especially closer to the fovea (right half of image). Boxed area is shown in Fig. 7. (**b**) Same image as (**a**), marking 826 absent outer segments with yellow dots and 124 intact outer segments with red dots. The region measures only .0903 mm^2^, because of the blood vessel, yielding a density of 10,520 cones/mm^2^ (9,147 missing cones/mm^2^ and 1,373 intact cones/mm^2^). (**c**) Same region, a year later, showing partial recovery in cone population. (**d**) Same image as (**c**), marking 432 absent outer segments (yellow dots) and 532 intact outer segments (red dots), for a density of 10,675 cones/mm^2^ (4,784 missing cones/mm^2^ and 5,891 intact cones/mm^2^). **(e)** For comparison, right retina centered at 6.5° nasal to the fovea, showing normal cone population (see green box, Fig. S6) in a region measuring 0.10 mm^2^ at the same eccentricity as the left retina. (**f**) Same image as (**e**), marking 1,229 visible outer segments (red dots) and 46 gaps (yellow dots). This corresponds to a density of 12,750 cones/mm^2^ (12,290 visible cones/mm^2^ and 460 presumptive cones/mm^2^.

In the nasal retina, the cones were affected far more severely. The montage of AOSLO images showed progressive depletion of outer segments as the optic disc was approached (Fig. 3, Fig. S2). At 6.5° eccentricity, there was an abrupt decrement in outer segment density, coinciding with the border of the scotoma surrounding the blind spot. The retina in a sample region, at the same eccentricity as the location analyzed in the left temporal retina, resembled an empty honeycomb with only a scant population of surviving outer segments (Fig. 5a,b). From 6.5° to the optic disc border there was dropout of more than 90% of cone receptors (Fig. 3, Fig. S2).

Over many months the subject’s retinal function gradually recovered, with shrinkage of the enlarged blind spot (Fig. 1b,c, and Fig. S3). AOSLO imaging was repeated one year later (Fig. S4). Many of the previously dark gaps in the cone mosaic were now filled by wave guiding cones with inner and outer segments. For example, in the sample field centered nasally at 6.5°, the density of intact cone photoreceptors increased from 1,373/ mm^2^ to 5,891/ mm^2^ (Fig. 5c,d). For comparison, the density of cones was determined at the same location in the unaffected right nasal retina (Fig. 5e,f, Fig. S6). This measurement yielded a visible cone density of 12,290/ mm^2^. Thus, in the left eye nearly 50% of the cone outer segments were still missing at 6.5° nasal to the fovea. Nonetheless, retinal sensitivity was reduced by only 3 dB (Fig. S3).

**Figure 6:**
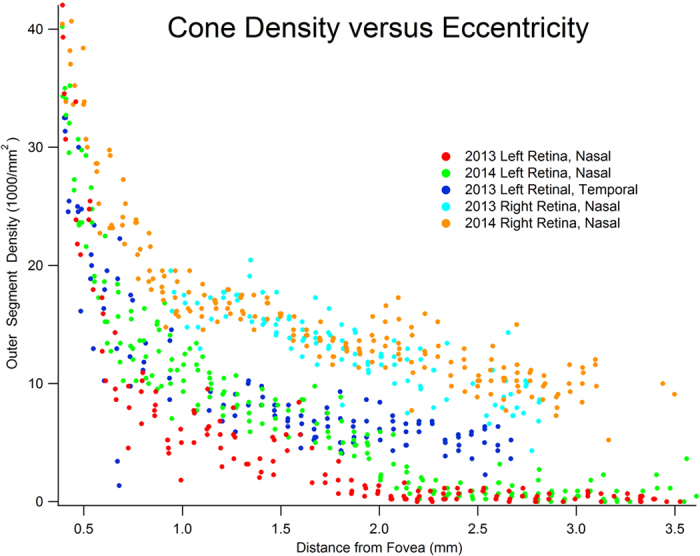
Graph of outer segment density as a function of distance from the fovea shows partial recovery of outer segment population in the left eye. Each point represents cone density at a local region within the AOSLO montage. All curves show the expected decline in cone density with increasing eccentricity (no data are shown within 400 μm of the fovea, because cones were too small to count reliably). The main finding is that nasal retina outer segment density increased in the left eye between 2013 and 2014 (p < 0.005, K-S test). The recovery was most evident from 1.0–2.5 mm, which correlated with shrinkage of the enlarged blind spot. Although cone loss was greatest in nasal retina near the optic disc, in 2013 outer segment density was also reduced in the temporal retina of the left eye. This region was not reimaged in 2014, so comparison was not possible. The outer segment densities in the right eye were equal in 2013 and 2014 (p = 0.479, K-S test), and matched reported values^31^.

**Figure 7:**
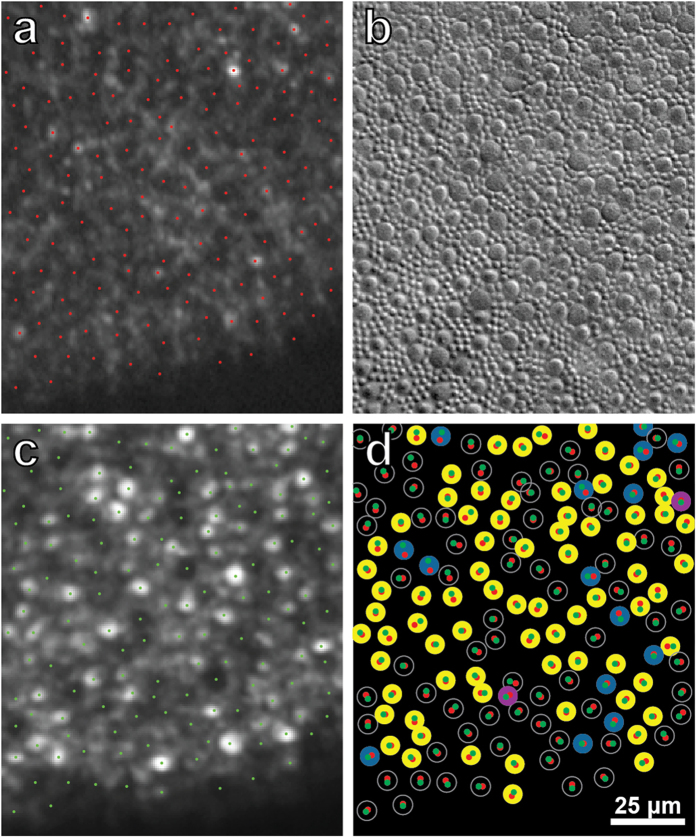
Regeneration of cone outer segments occurs at sites of previously missing outer segments. **(a)** Boxed region in Fig. 5a, marking the location of surviving and missing outer segments (red dots). (**b**) Cone and rod (smaller profiles) outer segments viewed in Nomarski optics in retina obtained from a normal subject, 6.5° nasal to the fovea. Spacing and size resemble missing cone outer segments and intact rod outer segments in (**a**). **(c)** Same field as in (**a**), imaged one year later. Many dark gaps have been replaced by new cone outer segments (Supplementary movie). **(d)** Locations of cone outer segments in (**a**) (red dots) and (**c**) (green dots) are paired, indicating that one can make a direct match between photoreceptor sites imaged one year apart. 69 unfilled circles = outer segments invisible in both 2013/2014; 15 blue circles = outer segments visible in both 2013/ 2014; 2 purple circles = outer segments visible in 2013/invisible in 2014; 74 yellow circles = outer segments invisible in 2013/visible in 2014. These 74 yellow circles represent outer segments which regenerated.

Quantification of cone density from fovea to optic disc confirmed that recovery of visual function in the left eye was associated with a significant increase in the number of cone outer segments from 2013 to 2014 (p < 0.005, K-S test) (Fig. 6). There are two potential mechanisms that might explain this increase. It is possible that the new outer segments belonged to new cones, generated *de novo*. Alternatively, surviving cones may have simply regenerated their outer segments. In the former case one would expect new outer segments to appear anywhere in the cone mosaic, whereas in the latter case one would expect a tight spatial correspondence between the empty silhouettes representing degenerated outer segments in 2013 and the bright profiles of new outer segments in 2014. To address this point, the AOSLO images obtained a year apart were carefully compared. Missing cone outer segments appeared as dark circles about 8 μm in diameter. They were surrounded by palisades of bright rod outer segments, each 2 μm in diameter (Fig. 7a). This interpretation of the AOSLO data was supported by Nomarski microscopy of the outer segments in a retina obtained post-mortem from a 37-year-old woman, showing cones and rods with similar size, spacing, and density at the same eccentricity (Fig. 7b). Interestingly, cones were decimated but rods appeared to be relatively unaffected in AOSLO images from our subject. This would imply that rods may be spared in AIBSE, a finding that one could confirm by performing scotopic perimetry.

In the AOSLO image acquired in 2014, half the missing outer segments were still missing (Fig. 7c). However, the remaining dark gaps in the photoreceptor mosaic had been filled in with new outer segments. Whether an outer segment was still missing (dark gap) or replaced (bright profile), there was a one-to-one match between each photoreceptor location in images taken before and after visual recovery (Fig. 7d). This observation implies that the location of cones remained static over a year, and consequently, that a cone which survived damage inflicted by the original disease process was able to generate a new outer segment.

**Figure 8:**
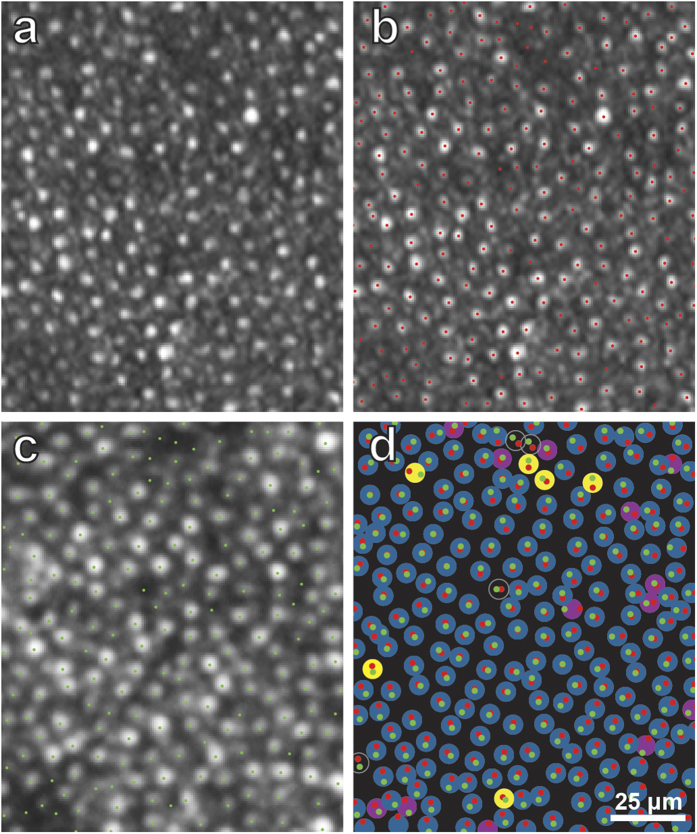
Stable alignment of cone outer segments in normal right retina over one year. **(a)** Boxed region in Fig. S5, showing cone mosaic in 2013. (**b**) Same image as (**a**), marking the location of each outer segment profile (red dots). **(c)** Same field as in (**a**), but imaged in 2014 (boxed region in Fig. 5f and S6), showing each outer segment profile (green dots). In (**a**), the imaging plane is slightly closer to the pigment epithelium than in (**c**), accounting for the more punctate appearance of the cones in (**a**). **(d)** Red and green dots, transferred from (**b**) and (**c**) respectively, showing that they form pairs. This indicates stable position of cone outer segments between 2013 and 2014. 4 unfilled circles = outer segments invisible in both 2013/2014; 206 blue circles = outer segments visible in both 2013/ 2014; 13 purple circles = outer segments visible in 2013/invisible in 2014; 6 yellow circles = outer segments invisible in 2013/visible in 2014. 90% of outer segments (blue circles, 206/229) appear visible in 2013 and 2014.

As a control, it is useful to compare the stability of cone locations in normal retina over the course of a year. Cone profiles were plotted in the right eye (Fig. 8), in a region matching the size and location of the field analyzed in the left eye (Fig. 7). About 90% of the outer segments matched in 2013 and 2014. Only a small number of outer segments seen in 2013 were invisible in 2014, and vice versa. This comparison indicates that outer segments are highly stable in normal retina, and that the large increase in visible outer segments that occurred from 2013 to 2014 in the left retina represents photoreceptor regeneration, rather than normal photoreceptor turnover.

## Discussion

Loss of cones has been shown with AOSLO in numerous retinal diseases^15^. In many forms of retinitis pigmentosa, post-mortem studies have confirmed that photoreceptors undergo pathological degeneration^16^. In such cases, there seems little doubt that cones appear absent in AOSLO images because they are no longer present physically^17^. It is more difficult to interpret images from conditions for which histological correlation is not available, such as AIBSE.

The problem is compounded by the fact that individual cones undergo changes in reflectance over time, and therefore, for any given cone, one cannot assume that absence of light back scatter means that the outer segment is missing^18^. This phenomenon probably explains why, even in our subject’s normal right retina, a small number of cones appeared absent in 2013 (yellow circles, Fig. 8d) or in 2014 (purple circles, Fig. 8d; yellow dots, Fig. 5f). Such cones are not being lost or regenerated, but simply undergoing spontaneous changes in AOSLO visibility. These fluctuations add some noise to observations of global changes in the cone outer segment population.

Recent evidence suggests that the reflectance of individual cones oscillates in a cyclical fashion, perhaps in relationship to disc shedding^19^. Serial imaging has shown that while some cones are becoming brighter, others are becoming dimmer. The percentage of cones that appears dark at any moment remains fairly constant over time. For the entire cone population, less than 6% of outer segments are too dark to identify reliably in any given image^20^. Accordingly, the apparent absence of 90% of outer segment profiles near the optic disc (Fig. 5a), suggested by the dark holes observed where cones should be present, cannot be ascribed simply to periodic changes in cone reflectance. However, such fluctuations in reflectance can explain why some cone profiles that were visible when our subject was first imaged were not apparent on follow-up imaging a year later, despite an overall recovery in cone density (e.g., those denoted by 2 purple circles in Fig. 7d).

The most likely interpretation of our findings is that outer segments were destroyed in our subject, resulting in the dark gaps in the initial AOSLO images. This inference is supported by optical coherence tomography (Fig. 2), which showed thinning and disruption of the outer segment layer, occurring only in severely affected retina near the optic disc. If acute idiopathic blind spot enlargement were caused by a process that left intact the structure of outer segments, but affected only their AOSLO reflectivity, one would not expect to see morphological changes with optical coherence tomography. It may seem puzzling that destruction of the outer segment layer imaged with optical coherence tomography was not more profound, given the devastation of the cone outer segments near the optic disc (Fig. 5a). However, in AOSLO images there was relative sparing of the rods, which may explain why the outer segment layer was partially preserved. In future studies, a split-detector could be used to image inner segments and outer segments at the same location, to garner further evidence that new outer segments can regenerate from surviving inner segments^13^.

After regeneration, the left retina contained about 50% of the normal concentration of outer segments at 6.5°, yet sensitivity to light was nearly normal, being reduced by only 3 dB. Relative sparing of light sensitivity, despite cone loss, has been reported previously in color blindness. For example, in deuteranopia a 30% loss of cones produces no visual field depression, unless gaps in the cone mosaic are probed with extremely small test spots^21^. It appears that the 0.43° spot used in standard clinical perimetry stimulates enough cones, even when their density is reduced sharply, that retinal sensitivity is only mildly affected. This observation implies that in various retinal diseases, a relatively modest recovery in the cone population may be enough to yield a substantial improvement in function. By the same token, staving off photoreceptor loss to maintain the cone population above a certain minimum density may be critical for preserving vision^22^,^23^.

The retina is susceptible to mechanical injury, from blunt trauma (commotio retinae), subretinal hemorrhage, or retinal detachment. With re-apposition of the retina to the pigment epithelium and the passage of time, there is usually slow improvement in visual function. Regeneration of outer segments may contribute to this recovery, but would be difficult to show using AOSLO, because of the difficulty of obtaining satisfactory baseline images of a retina that has been disrupted physically. In a disease such as AIBSE, the situation is auspicious for AOSLO imaging, because vision loss does not follow a mechanical insult to the retina.

In an animal model of hereditary retinal disease, ciliary neurotrophic factor has been shown to reverse the degeneration of outer segments^24^. In humans with retinitis pigmentosa, AOSLO imaging has shown that ciliary neurotrophic factor can slow the rate of outer segment loss^23^. With this report, we provide direct morphological evidence, based on before-and-after imaging of the same retina, that human outer segments can regenerate spontaneously. Normal photoreceptor outer segments undergo incremental renewal on a daily basis to replace membrane discs that are shed from their tips^25^,^26^. The spontaneous restoration of outer segments in our subject may have harnessed this intrinsic mechanism, much like a new toenail can grow after one is lost from trauma. Determining the mechanisms that control outer segment renewal, and learning how to activate this process when necessary, may be useful for treatment of vision loss incurred by loss of outer segments^27^.

## Methods

### Subject Data

All research procedures were approved by the Committee on Human Research at the University of California San Francisco. The subject provided informed written consent for participation. All examinations and tests were carried out in accordance with the approved guidelines. The subject was an 18-year-old woman in excellent health who developed a spontaneous temporal visual field defect in her left eye from a syndrome known as acute idiopathic blind spot enlargement. She had no other history of illness, hospitalization, or treatment with any medication. Her visual acuity was 20/15 in each eye and there was no refractive error. An afferent pupil defect was present in the left eye. Slit lamp examination and fundus examination with a direct ophthalmoscope were normal. A fluorescein angiogram was also normal (not illustrated). After her initial examination, the patient was seen periodically over the next year to monitor her recovery.

### Visual Field Testing

Automated perimetry was performed with a Humphrey Field Analyzer (Carl Zeiss Meditec, Dublin, CA) using a full threshold algorithm and a size III (0.43°) white stimulus. No refractive correction was necessary. On each occasion, the central 30° and the peripheral 30–60° of the visual field were tested in each eye.

### Optical Coherence Tomography

A Heidelberg Spectralis (Heidelberg Engineering, Carlsbad, CA) instrument was used to obtain spectral domain optical coherence tomographic (OCT) images of each retina, both at the time of her initial evaluation and each follow-up visit.

### Multifocal Electroretinography

A multifocal electroretinogram (mfERG) was obtained using a VERIS Clinic 5.1.10X model instrument (Electro-Diagnostic Imaging, Redwood City, CA), following standards of the International Society for the Clinical Electrophysiology of Vision^28^. The subject’s pupils were dilated with 1% tropicamide and 2.5% phenylephrine hydrochloride. She was in a light-adapted state. A Burian-Allen contact lens electrode was applied. The stimulus consisted of 103 hexagons subtending the central 40° of the visual field. Their mean luminance was 100 cd/m^2^, with light tiles measuring 200 cd/m^2^ and dark tiles 4 cd/m^2^. An infrared camera was used to monitor fixation. Responses were recorded during 16 sequences, each lasting 30 sec. The signal was amplified by 10^5^, band-pass filtered between 10–100 Hz, and spatially averaged at 17%.

### Adaptive Optics Scanning Laser Ophthalmoscopy

To probe the cause of visual field loss in acute idiopathic blind spot enlargement, AOSLO was used to acquire images of the photoreceptor mosaic after pharmacological dilation of the pupils. Both imaging sessions were conducted in the early afternoon. The subject’s head was stabilized in a chin/forehead rest while she fixated a green 0.20° laser target spot. The imaging system consisted of a low-coherence, 840-nm light source, a Shack-Hartman wavefront sensor, and a 140-actuator microelectromechanical deformable mirror. A 10 sec, 300 frame, 512 × 512 pixel video subtending approximately 1.2 × 1.2° was recorded at each location, beginning in the fovea. Subsequent videos were recorded by displacing the fixation target along the horizontal meridian of the visual field in 0.5–1.0° steps. Image offset and warp, which occur from frame to frame due to fixation instability, were corrected with custom software employing a map-seeking circuit algorithm^29^. The individual frames comprising each video were inspected to eliminate occasional frames that were marred by blinks or other artifacts (Virtual Dub, www.virtualdub.org). The remaining frames were averaged to maximize the signal-to-noise ratio (Matlab, Mathworks, Natick, MA). The final image was assembled into a montage by translation and rotation to match overlapping features in adjacent images. The resulting montage was then positioned on a photograph of the retina by using blood vessels for alignment (Photoshop CS6, Adobe Systems, San Jose, CA).

To measure cone density, a grid was superimposed on each montage to divide it into tiles measuring 100 × 100 pixels (420 pixels/deg). Tiles that contained a portion of a blood vessel which obscured the cone mosaic were eliminated. In addition, tiles that extended beyond the edge of the montage were eliminated. The number of cones in each of the remaining tiles was counted manually. The identification of cone outer segments, and the empty silos left by missing outer segments, depended on observer judgement. All the AOSLO images used to compile the measurements of cone density in Fig. 6 are available in Fig. S2, 4, 5, and 6. In addition, AOSLO images and the corresponding designations of intact versus missing outer segments are shown as separate images (e.g., compare Fig. 5a–f), so that one can form one’s own judgment regarding the accuracy of outer segment profile identification.

A total of 773 tiles was analyzed and 36,141 cones were counted. The density of cones was calculated for each tile. The result was plotted for each montage as a function of distance from the fovea (Fig. 6) The distance from the center of each tile to the fovea was determined by using 280 μm/deg as a conversion factor^30^. Plots of cone density as a function of eccentricity were analyzed statistically by comparing pairs of data sets (e.g., 2013 left nasal retina versus 2014 left nasal retina) using the Kolmogorov-Smirnov test. The plots were converted to cumulative distributions of tile cone density to calculate a D statistic. The resulting p value provided a measure of the probability that the difference observed in cumulative frequency distributions would occur by chance.

### Retinal Histology

The eyes from a 37-year-old woman with normal visual function who died from idiopathic pulmonary fibrosis were removed 6 hours after death and placed into 4% paraformaldehyde. Each retina was dissected from the pigment epithelium and flat-mounted onto a glass slide, with the photoreceptor layer facing up. The retina was cleared with dimethyl sulfoxide and coverslipped with glycerin. Stacks of images of photoreceptor processes were obtained at different eccentricities between the fovea and optic disc in each eye using an inverted research microscope (Nikon Eclipse Ti) equipped with Nomarski interference contrast optics. Each image had a depth of field of 1.44 μm and individual images were obtained every 0.5 μm in the Z-plane. Imaging from the tips of the photoreceptors to the external limiting membrane yielded about 70 images, about half passing through outer segments and half through inner segments.

## Additional Information

**How to cite this article**: Horton, J. C. *et al.* Spontaneous Regeneration of Human Photoreceptor Outer Segments. *Sci. Rep.* **5**, 12364; doi: 10.1038/srep12364 (2015).

## Supplementary Material

Supplementary Information

Supplementary Movie 1
