# Supplementary material for: Spontaneous Regeneration of Human Photoreceptor Outer Segments
Source: Sci Rep. 2015 Jul 27;5:12364. doi: 10.1038/srep12364 (PMC4515765; doi:10.1038/srep12364)
Supplement: Supplementary Information [file srep12364-s1.pdf]

SUPPLEMENTARY FIGURES AND LEGENDS

FOR PAPER:

SPONTANEOUS REGENERATION OF HUMAN PHOTORECEPTOR OUTER SEGMENTS

Jonathan C. Horton, Alicia B. Parker, James V. Botelho, Jacque L. Duncan

Beckman Vision Center
University of California San Francisco
San Francisco, California, 94143, USA

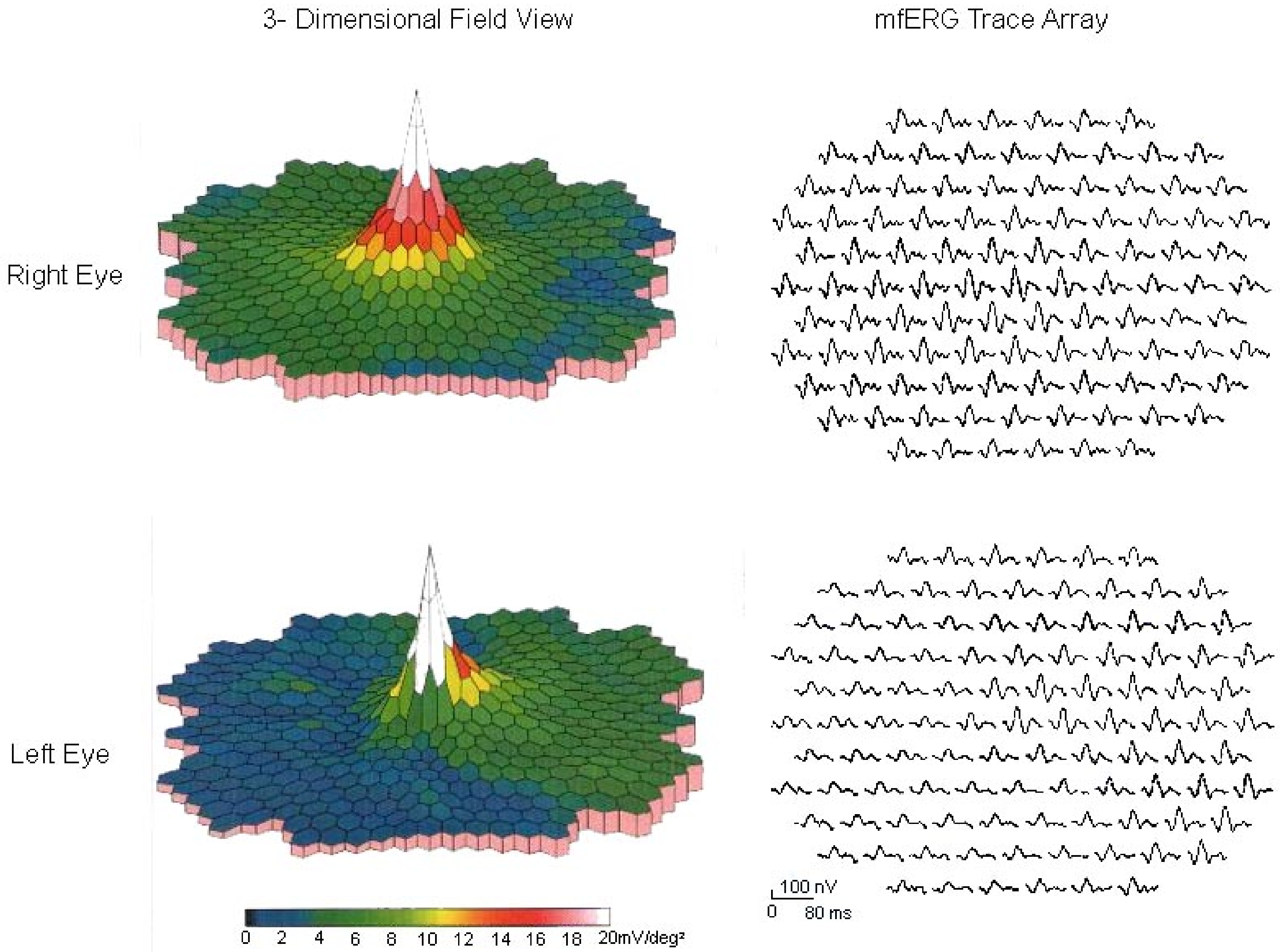

3- Dimensional Field View
mfERG Trace Array
Right Eye
Left Eye
100 nV
0
80 ms
0 2 4 6 8 10 12 14 16 18 20mV/deg²

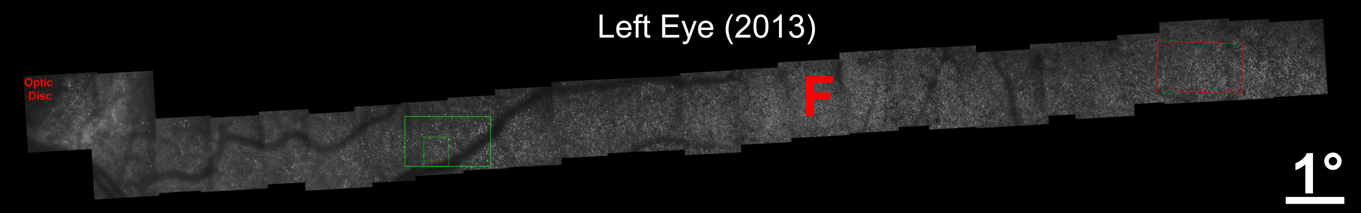

Left Eye (2013)
Optic Disc
F
1°

A Initial Left Eye Visual Field - Central 30°

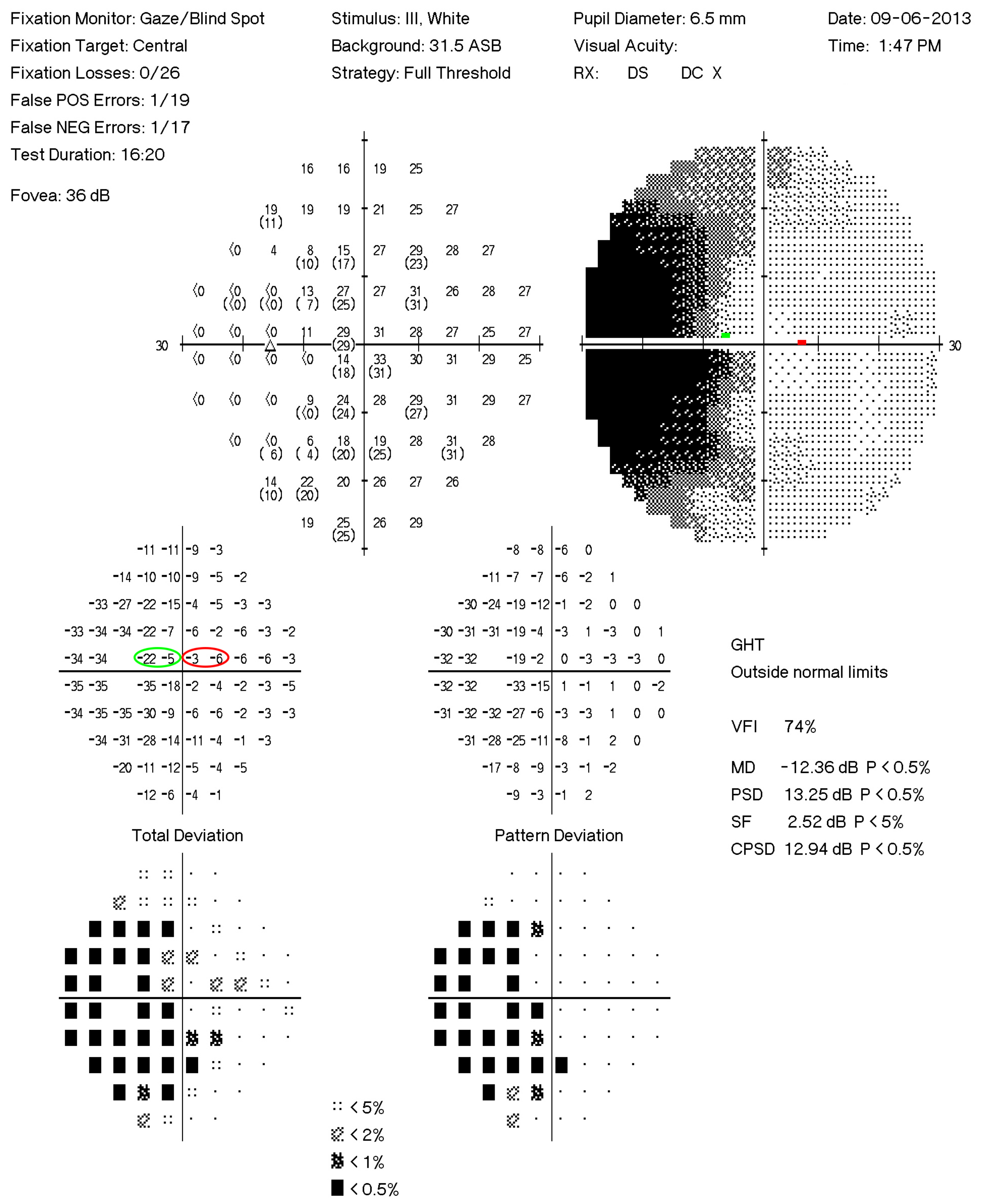

B Initial Left Eye Visual Field - Peripheral 30 - 60°

Fixation Monitor: Gaze/Blind Spot
Fixation Target: Central
Fixation Losses: 0/12
False POS Errors: 0 %
False NEG Errors: 0 %
Test Duration: 04:11

Fovea: OFF

Stimulus: III, White
Background: 31.5 ASB
Strategy: SITA-Fast

Pupil Diameter: 6.4 mm
Visual Acuity:
RX: DS DC X

Date: 09-06-2013
Time: 2:45 PM

Threshold Graytone

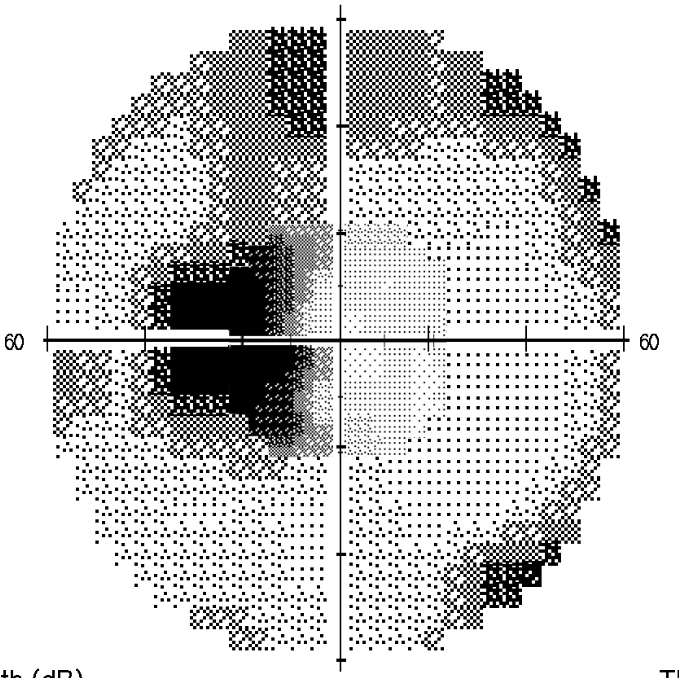

Defect Depth (dB)

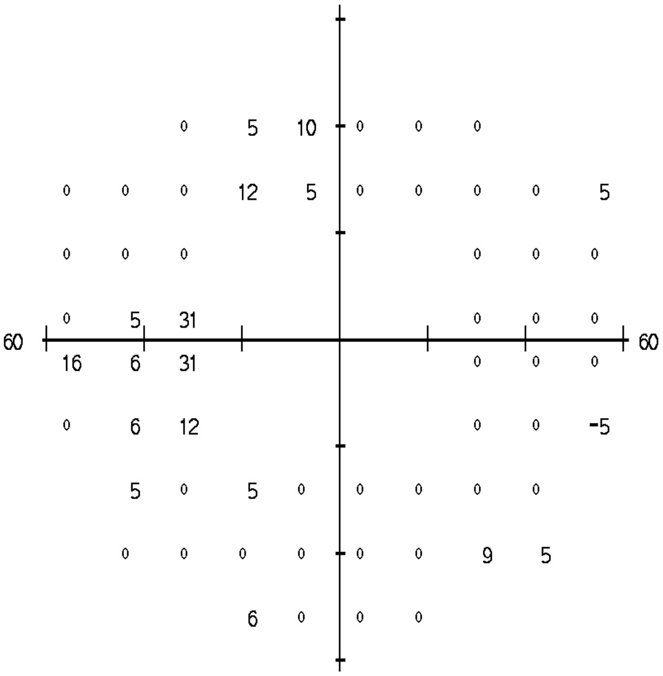

Threshold (dB)

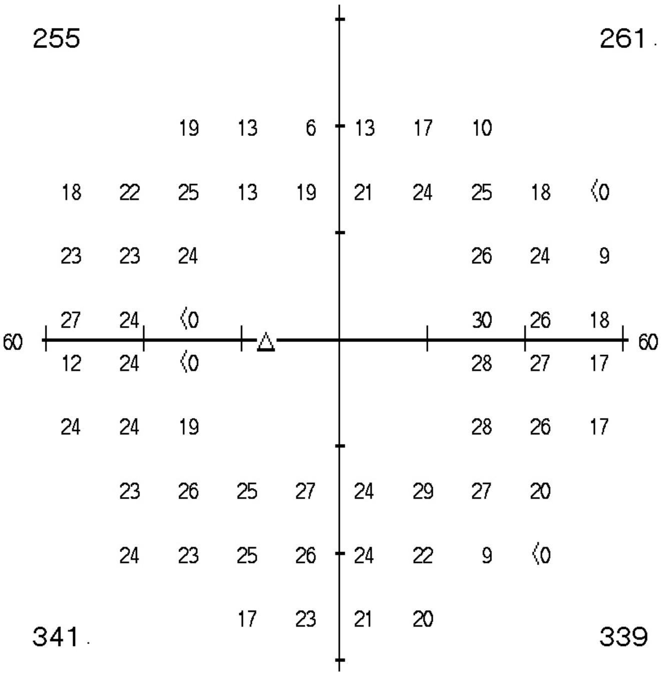

o = Within 4 dB of Expected
Central Reference: 37 dB

C Initial Right Eye Visual Field - Central 30°

Fixation Monitor: Gaze/Blind Spot
Fixation Target: Central
Fixation Losses: 6/23 xx
False POS Errors: 1/15
False NEG Errors: 0/14
Test Duration: 12:40

Fovea: 38 dB

Stimulus: III, White
Background: 31.5 ASB
Strategy: Full Threshold

Pupil Diameter: 6.6 mm
Visual Acuity:
RX: DS DC X

Date: 09-06-2013
Time: 1:31 PM

		29	27		25	29			
	29	29	29		30	28	30		
29 (27)	30	27 (29)	31		30	29 (31)	31	31	
27	28	26	31 (31)	29	34	29 (29)	30	32	30
27	29	29	30	33	40 (34)	30	29	29	31
28	30	30	33	36 (34)	34 (30)	32	2	31	31
26	30	32	31 (31)	31	35	31 (33)	31	31	27
	27	28 (28)	29	30	30	32	29 (31)	31	
		31	26	31	29	31	31		
			30	29	32	32			

30

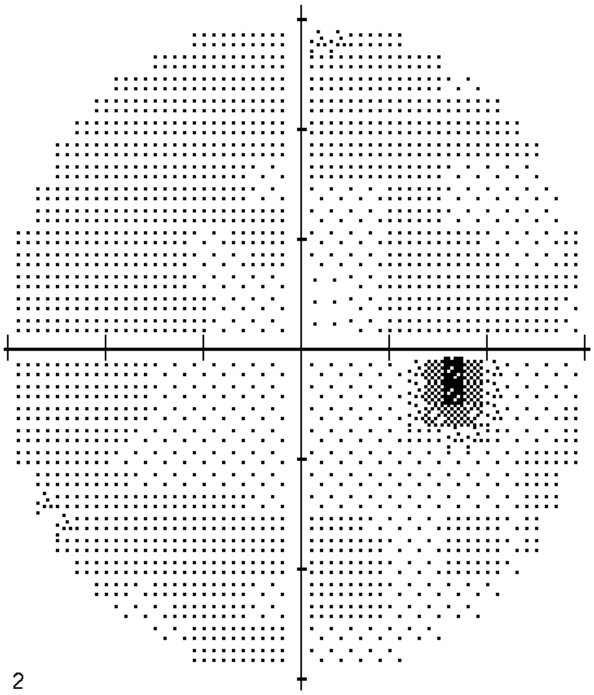

			1	-1	-2	2			
		0	-1	-1	1	-1	1		
	-2	-1	-3	0	-1	-1	0	0	
-2	-3	-6	-2	-4	1	-3	-2	0	-1
-3	-2	-4	-4	-1	3	-3		-3	-1
-2	-2	-3	-1	1	-2	-1		-2	-2
-4	-2	-1	-3	-3	2	-1	-2	-2	-5
	-4	-4	-3	-3	-3	-1	-3	-1	
		0	-5	0	-3	-1	-1		
			0	-1	1	1			

Total Deviation

			1	-1	-2	2			
		0	-1	-1	0	-1	1		
	-2	-1	-3	0	-1	-1	0	0	
-3	-3	-6	-2	-4	1	-3	-2	0	-2
-3	-3	-4	-4	-1	3	-3		-4	-2
-3	-2	-4	-1	0	-2	-2		-2	-2
-4	-2	-1	-3	-3	1	-1	-2	-2	-6
	-4	-4	-4	-3	-3	-1	-3	-2	
		0	-5	-1	-3	-1	-1		
			0	-1	1	1			

Pattern Deviation

GHT
Within normal limits

VFI 100%

MD -1.68 dB
PSD 2.08 dB
SF 1.37 dB
CPSD 1.38 dB

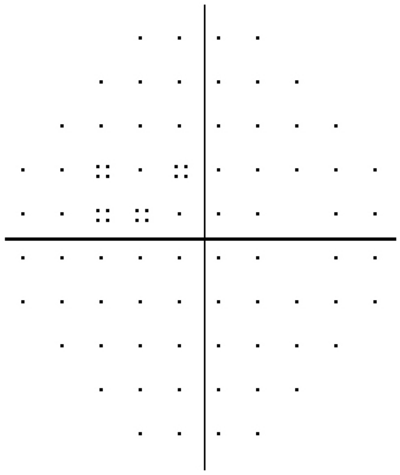

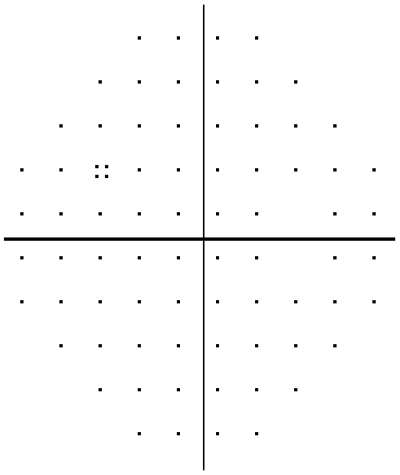

:: < 5%
< 2%
< 1%
< 0.5%

D Left Eye Visual Field - 3 Months Later

Fixation Monitor: Gaze/Blind Spot
Fixation Target: Central
Fixation Losses: 0/14
False POS Errors: 0 %
False NEG Errors: 5 %
Test Duration: 04:43

Fovea: 37 dB

Stimulus: III, White
Background: 31.5 ASB
Strategy: SITA-Fast

Pupil Diameter: 6.4 mm
Visual Acuity:
RX: DS DC X

Date: 12-11-2013
Time: 1:16 PM

			22	25	28	28			
		18	25	24	29	29	23		
	24	24	27	26	30	30	29	28	
13	21	26	26	29	30	31	29	28	24
13	21	0	11	30	31	30	30	27	27
19	8	<0	24	30	30	31	31	27	27
13	16	<0	25	29	29	30	30	29	27
	25	20	18	29	24	27	29	28	
		31	30	29	25	28	27		
			27	29	26	26			

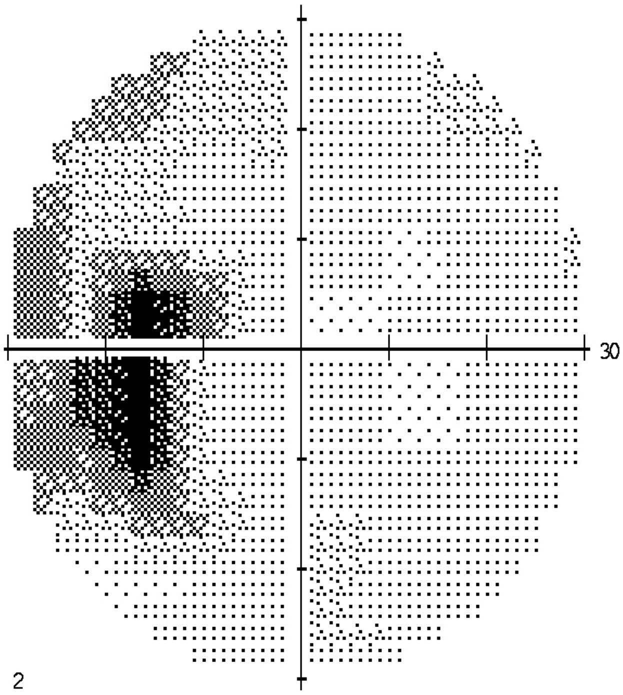

			-6	-3	0	-1			
		-12	-5	-6	-1	-1	-7		
	-8	-7	-5	-6	-3	-2	-3	-3	
-19	-11	-7	-7	-4	-4	-3	-4	-3	-6
-19	-11		-23	-5	-4	-5	-4	-6	-4
-13	-24		-11	-5	-6	-4	-3	-5	-3
-19	-16	-35	-9	-6	-5	-4	-3	-3	-3
	-8	-12	-15	-5	-9	-6	-3	-3	
		-2	-2	-3	-7	-3	-4		
			-5	-2	-4	-4			

			-3	0	3	2			
		-9	-2	-3	1	1	-5		
	-5	-4	-2	-3	0	1	0	0	
-16	-8	-4	-4	-2	-1	0	-1	-1	-4
-17	-9		-20	-2	-1	-2	-1	-3	-1
-10	-22		-8	-3	-3	-1	0	-2	-1
-16	-14	-32	-6	-3	-2	-2	-1	0	0
	-5	-9	-13	-2	-6	-3	-1	0	
		1	0	0	-4	-1	-1		
			-2	1	-2	-1			

GHT
Outside normal limits

VFI 90%

MD -6.16 dB P < 1%
PSD 5.44 dB P < 0.5%

Total Deviation

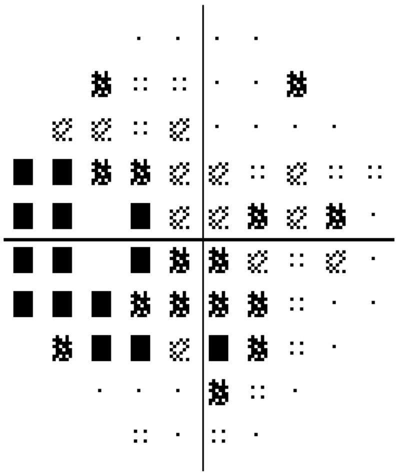

Pattern Deviation

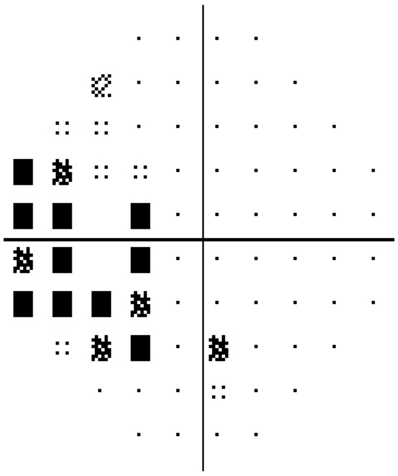

< 5%
< 2%
< 1%
< 0.5%

E

Left Eye Visual Field - 1 Year Later

FIXATION MONITOR: BLIND SPOT
FIXATION TARGET: CENTRAL
FIXATION LOSSES: 0/12
FALSE POS ERRORS: 0 %
FALSE NEG ERRORS: 0 %
TEST DURATION: 03:40

FOVEA: 39 DB

STIMULUS: III, WHITE
BACKGROUND: 31.5 ASB
STRATEGY: SITA-FAST

PUPIL DIAMETER:
VISUAL ACUITY:
RX: DS DC X

DATE: 08-19-2014
TIME: 1:19 PM

			26	24	27	28			
		28	28	28	28	29	29		
	29	29	29	29	29	30	30	30	
24	27	29	29	31	32	32	30	30	29
27	26	12	31	33	34	32	30	30	27
26	28	<0	27	32	32	34	33	32	29
26	29	28	30	32	32	33	32	32	28
	30	28	31	31	31	33	32	32	
		30	31	31	31	31	29		
			30	30	31	31			

30

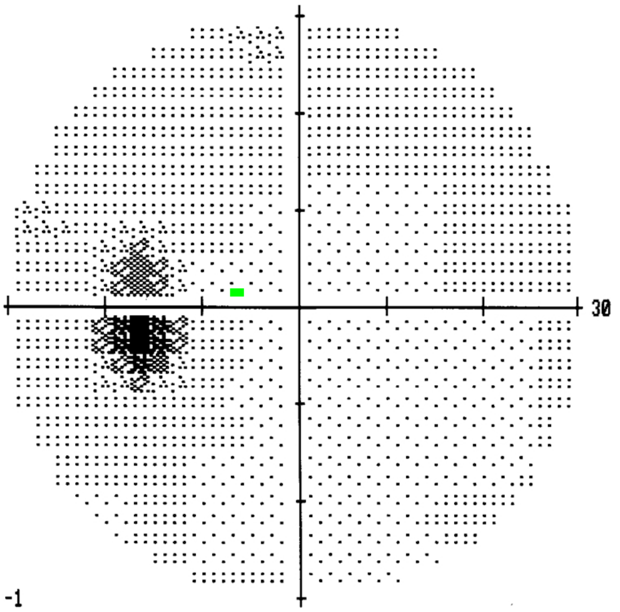

			-2	-4	-2	-1			
		-2	-2	-2	-2	-2	-2		
	-3	-3	-3	-3	-3	-3	-2	-1	
-8	-5	-3	-4	-3	-1	-2	-3	-2	-1
-5	-6		-3	-2	-1	-3	-3	-2	-3
-5	-4		-8	-3	-3	-1	0	0	-1
-5	-3	-5	-4	-2	-2	-1	-1	0	-2
	-2	-5	-2	-2	-2	0	0	1	
		-2	-1	-1	0	0	-1		
			-2	-1	1	1			

			-2	-4	-1	-1			
		-1	-1	-1	-1	-1	-1		
	-2	-2	-2	-2	-3	-2	-1	-1	
-8	-5	-3	-3	-2	-1	-1	-2	-1	0
-4	-6		-3	-2	-1	-2	-3	-2	-3
-5	-4		-7	-2	-2	-1	0	0	-1
-5	-3	-5	-3	-2	-2	0	-1	1	-1
	-1	-4	-2	-1	-2	1	0	1	
		-2	-1	0	0	0	-1		
			-1	0	1	1			

GHT
WITHIN NORMAL LIMITS

MD -2.19 DB P < 5%
PSD 1.86 DB

TOTAL
DEVIATION

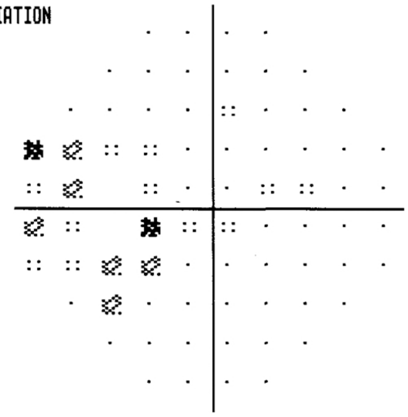

PATTERN
DEVIATION

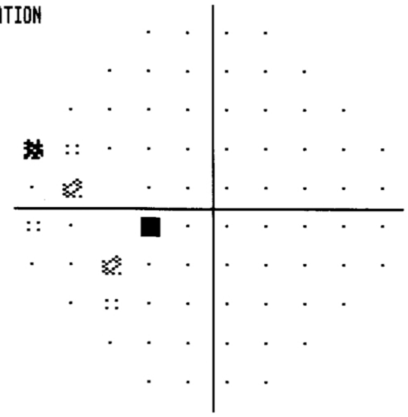

:: < 5%
< 2%
< 1%
■ < 0.5%

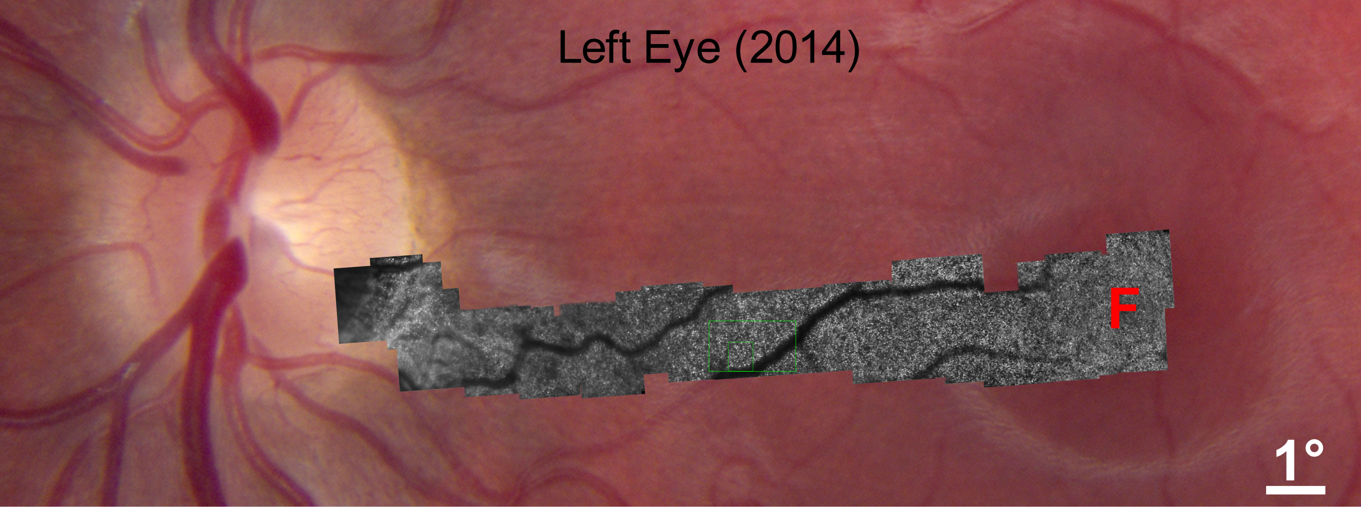
Left Eye (2014)
F
1°

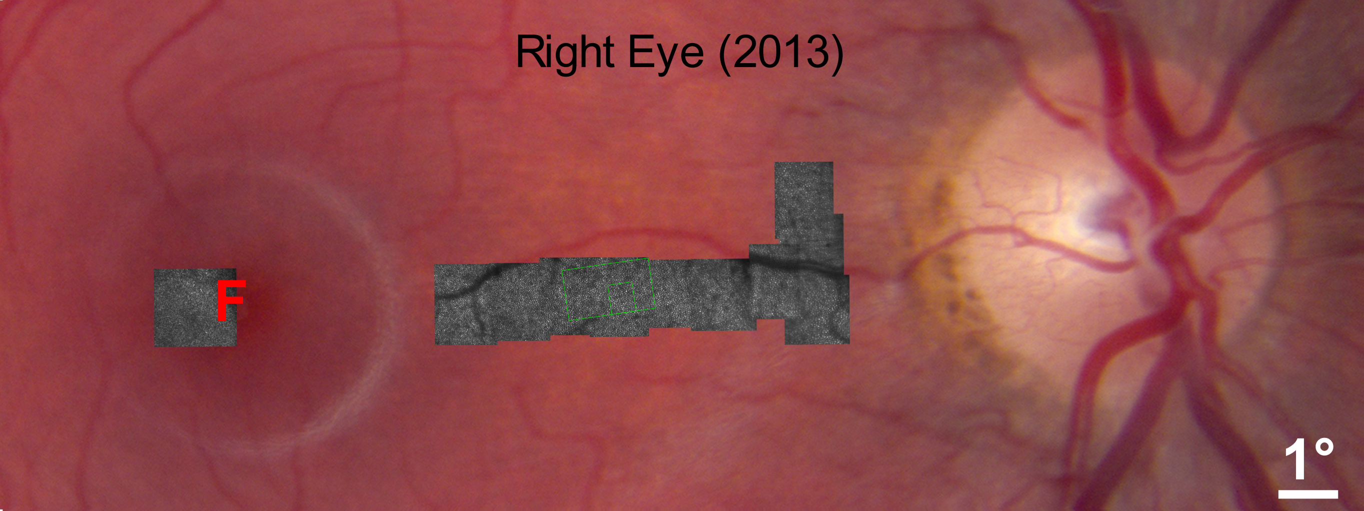
Right Eye (2013)
F
1°

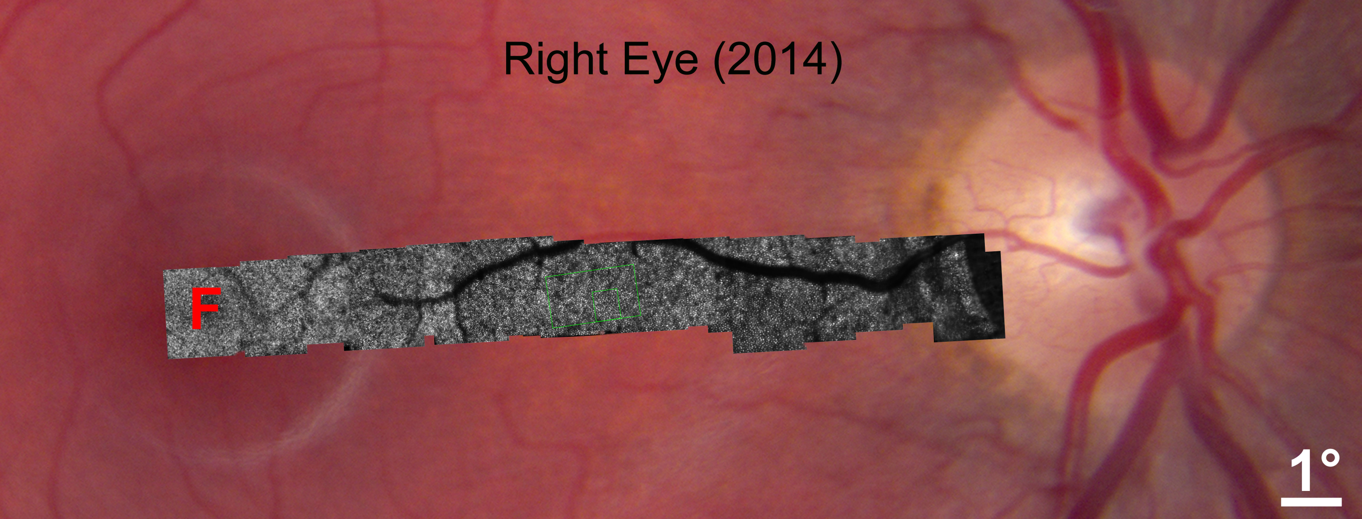
Right Eye (2014)
F
1°

Supplementary Figure Legends

Figure S1) Multifocal electroretinogram (mfERG) shows reduced response density of traces from the nasal retina of the left eye, corresponding to the subject's visual field scotoma. The 3-dimensional plot shows a normal foveal peak, but diffuse depression beginning 5° from the center of gaze (blue tiles). The mfERG of the right eye is normal.

Figure S2) AOSLO montage from **Fig. 3**, showing the cone mosaic at higher resolution. Red rectangle appears in **Fig. 4**; green rectangle in **Fig. 5a**. F = fovea.

Figure S3) Full printouts of Humphrey visual field data, from which **Fig. 1** was derived. **(a)** Initial central 30° left eye visual field test showing a huge blind spot. Stimuli 0.43° in diameter are presented for 200 msec in random order at locations spaced every 6° within a hemispheric bowl against a background illumination of 31.5 apostilbs. Retinal sensitivity is determined using a full threshold strategy, reducing stimulus intensity by 4 dB increments until threshold is crossed, and then increasing intensity by 2 dB until threshold is recrossed. Numbers in grid at upper left are raw values in decibels (dB), signifying retinal sensitivity. A value of 0 dB indicates no response to the brightest spot (10,000 apostilbs) and a value of 40 dB indicates a response to a dim spot only 1 apostilb above the background. The grayscale representation at upper right is based on the numeric dB values, with black representing no response to the brightest spot. The green rectangle corresponds to the location in the visual field of the cone mosaic in **Fig. 5a**. The red rectangle corresponds to the location of in the visual field of the cone

mosaic in **Fig. 4**. Grids of negative numbers in middle of printout indicate the difference (dB) between the subject's thresholds and the expected thresholds for age-matched normal subjects. Pattern deviation plot is similar, but corrected for shift in overall field sensitivity. Green and red circles indicate defects at 3° and 9°, which straddle the location of green and red rectangles on grayscale map. The sampling grid of the perimeter is relatively coarse, so the border of the scotoma is not defined with precision. MD = mean deviation of tested points; PSD, SF, and CPSD indices are not relevant. **(b)** Initial peripheral 30-60° left eye visual field test, with the central 30° test from (**a**) inserted, showing that the scotoma in the left eye consists of an enormously enlarged blind spot. **(c)** Initial central 30° right eye test showing a normal visual field. Compare the blind spot with the blind spot in the left eye (**a**). **(d)** Left eye visual field shows some recovery 3 months later. **(e)** Left eye visual field a year later is improved, but the deviation plots (below) reveal residual blind spot enlargement. Retinal sensitivity is nearly normal in the region that bordered the former scotoma (green rectangle).

Figure S4) AOSLO montage of cones in the left eye, obtained a year after the initial imaging, when the blind spot had shrunken back to a more normal size (**Fig. S3e**). Many new cone outer segments have appeared, presumably accounting for diminution in the subject's scotoma. The green rectangle marks the area shown in **Fig. 5c**. F = fovea.

Figure S5) AOSLO montage of cones from the fovea (F) to the optic disc in the unaffected right eye obtained at the same time as the montage of decimated cones in the left eye (**Fig. 3, Fig. S2**). The cone outer segments are normal in appearance and density. Region enclosed by the small

green rectangle is shown in **Fig. 8a, b**. Large green rectangle corresponds to the zone shown in **Fig. S6** in 2014.

Figure S6) AOSLO montage in the right eye, obtained a year after the montage in the right eye shown in **Fig. S5**. There is a normal population of outer segments, with no change in density between the two montages. Region enclosed by the green rectangle is shown in **Fig. 5e**.

Supplemental Movie Legend

The movie compares the cone mosaic in **Fig. 7a** (2013 Left Retina) with the same region in **Fig. 7c** (2014 Left Retina). As the transition occurs back-and-forth between the two images, one can appreciate that about half of the empty cone profiles in 2013 become filled with bright outer segments in 2014.
